# Supplementary material for: Paramedic interactions with the packaging of medications and medical supplies: Poor package design has the potential to impact patient outcomes
Source: PLoS One. 2021 Aug 11;16(8):e0255099. doi: 10.1371/journal.pone.0255099 (PMC8357099; doi:10.1371/journal.pone.0255099)
Supplement: S1 File — (DOCX) [file pone.0255099.s001.docx]

**Packaging Survey Cognitive Testing Results – September 14, 2016**

**Background**

A draft version of the packaging survey items was revised after discussion between the project team. The revised survey instrument was then cognitively tested with practicing EMS providers.

The general goal of cognitive testing is determining whether survey items are functioning as intended. Specifically, probes and prompts are administered to provide insights into respondents’ cognitive processes, to ascertain:

(1) Is the item being comprehended as intended by the item writer?

(2) Is the respondent a knowledgeable informant? That is, does the respondent possess the knowledge needed to answer the question?

(3) Can respondents use the knowledge they possess to form a judgment (answer the question) in an appropriate manner?

(4) Will respondents with similar experiences select the same response option?

**Methods**

A protocol to enable cognitive testing was developed. Since none of these questions have been used previously, we needed to test the content and applicability to this specific population.

This protocol was administered to six EMS professionals from September 6-12. Cognitive interview participants included: an EMT in an ALS level service with 6 years of experience, an EMT at a fire-based service with unknown years of experience, a paramedic at a fire-based service with 12 years of experience, a paramedic at a fire-based 911 service with 8 years of experience, a paramedic at a private service with 5 years of experience, and a critical care paramedic at a private service with 7 years of experience. The protocol, along with responses to its probes and prompts, is attached.

Responses were reviewed and discussed by NREMT Research staff to identify potential problems.

**Results and Recommendations**

As is typical of cognitive testing, a variety of problems and potential problems were noted. These problems reflected several general areas of concern. They are discussed below, followed by item-specific problems not associated with these general areas. Suggestions for dealing with each of these concerns are also provided.

1. *Device versus medication.* Generally, participants had difficulty differentiating between a device and medication. While some participants assumed the terms referred to the same items, others mentioned “medical equipment” with specific examples of items such as IV catheters, laryngoscopes and the Res-Q-Pod. One participant initially thought the term “device” referred to implanted medical devices found in patients but eventually realized via context that the survey was requesting information about medical equipment found on the ambulance.

When asked if they had trouble with both a medication or device or just one, all participants initially responded that could only think of challenges with medications. Participants were able to list examples of devices, but all examples of recent challenges in identifying and opening packaging pertained to medications, with the exception of one packaging of a device used in conjunction with intubation called a PEEP valve. There was little differentiation in the survey responses and debriefing discussion between medications and devices, therefore if differences between the challenges of the two are important splitting the questions to ask about each separately may be needed.

**Recommendation: Change the question stems to “medical equipment” instead of devices or only ask about medications. If responses about devices are critical to the study, separating the questions would help elucidate differences in challenges between medications and devices.**

1. *Participant informant ability.* Participants were all found to be knowledgeable informants regarding usage of medication and devices. Even for participants who stated they had not had trouble opening medications or devices, they noted usage of these items every shift.

The debriefing participants included four paramedics and two EMTs. In most states, the scope of practice of an EMT limits their ability to use and dispense many medications and invasive medical devices. While the EMTs were able to understand the questions and provide examples of medications and devices, their responses may be limited due to a limited usage of said products.

Since paramedics have more opportunities with a wider range of medications and equipment, limiting the survey population to this provider level may yield more usable data.

**Recommendation: Limit survey population to paramedics to obtain the most useable data.**

1. *Adding a time period.* When participants were asked to articulate a specific example of situations in which they struggled with packaging challenges, we also asked them for when the last time this had happened. Most participants mentioned situations that were recent (within the past several weeks), however two mentioned examples taking place a year or two prior. Salient events are often recalled months to years later, however in general, recall bias can exist when asking about non-recent events. To limit this bias, we generally ask about a specific time period for respondents to reference, such as in the past 12 months.

**Recommendation: Rephrase the stems asking respondents if they have ever had difficulty to “In the past 12 months, have you had difficulty…”**

1. *Packaging challenges and coping mechanisms.* Participants were asked questions as to what other barriers and coping mechanisms they utilized to deal with packaging challenges. Generally, participants felt the barriers listed were comprehensive. We also tested to understand comprehension of some of the response options such as “materials meant to separate stick together.” Overall, no issues were found with comprehension.

Additional barriers to opening medications or devices mentioned by participants included specific product challenges such as using an Act-O-Vial or opening the plastic packaging of a PEEP valve. While these examples are specific to a certain product, the general theme is unfamiliarity with a packaging type. One participant recalled a problem with boxes that need to open via a specific end that is not well marked. No participant listed any additional barriers to identifying a medication.

When asked if there were any additional coping mechanisms they used to identify medications, two participants stated they would or have rearranged their medication bags/kits to prevent similar medications being stored close together. While the response option “position of product within container or the ambulance” does cover this coping action, no participant chose that option. Changing the response option to “changing location of product within container, bag or ambulance” may clarify the intention of this option.

**Recommendation: For the barriers to opening medications or device, add response options of a) Unfamiliar with product packaging and b) Packaging marking not clear. For coping mechanisms used to identify medications, change response option to read as “changing location of product within container, bag, or ambulance.”**

1. *Dispensing.* To check understanding of the term “dispensing a medical product,” participants were asked a series of questions including what products came to mind and what dispensing a medical product meant. Specific products noted included medications and their preparation and administration. Repeated themes included opening aspirin bottles and dealing with nitroglycerin tablets.

All participants described medication administration issues when asked what dispensing a medical product meant. These included selecting an IV catheter size that precluded easy use of a viscous medication such as Dextrose 50% or obtaining just one tiny nitroglycerin tablet from a full bottle.

Participants overwhelmingly answered that they did not have difficulties in dispensing a medical product. During the debriefing, several participants came up with some examples after discussion that they had not previously thought about. One participant suggested using the term “operating” a medical product or device if looking for responses other than medication administration.

**Recommendation: Change the question stem to either ask about medication administration or operating medical products/equipment depending on the purpose of the question.**

1. *Impact on patient care.* While the survey assesses the challenges respondents may potential face with packaging, no question relates the package challenges to impact of clinical care. One participant questioned if we were asking about situations where only a medication error occurred due to a packaging issue. While this is likely rare, linking the packaging issues to care would be important for key stakeholders in EMS.

**Recommendation: Add an item to assess the impact of packaging difficulties (identification, opening, and dispensing medical products) on patient care.**
