# Supplementary material for: Paramedic interactions with the packaging of medications and medical supplies: Poor package design has the potential to impact patient outcomes
Source: PLoS One. 2021 Aug 11;16(8):e0255099. doi: 10.1371/journal.pone.0255099 (PMC8357099; doi:10.1371/journal.pone.0255099)
Supplement: S3 File — (PDF) [file pone.0255099.s003.pdf]

Packaging

Thank you for agreeing to participate in this study! This project is a collaboration between the NREMT and Michigan State University's School of Packaging. Results of this survey will help us to better understand the barriers EMS professionals face in dealing with packaging in the prehospital environment and the coping strategies used to overcome these barriers.

The survey will take 5-10 minutes. Your participation in this research project is entirely voluntary. The NREMT does not mandate/require participation in this project, and as such there are no penalties associated with not participating or discontinuing participation at any time. You may refuse to answer any question by selecting 'Next' to move to the next question. Additionally, you can quit the survey at any time. Further, there are no foreseeable risks in participation.

Individuals who complete the survey by December 15, 2016, will be entered into a random drawing for one of ten $100 Amazon gift cards. Gift cards will be delivered electronically to the email address used to participate in the study. No personal information will be provided to Amazon.

Your privacy is important to us, and your responses will be kept absolutely confidential. Only data summarizing groups of participants will be reported. By clicking 'Next' below you are consenting to participate in the study. If you have any questions or want to obtain more information about this very important project, please contact the NREMT Research Department at 614-888-4484 or via email at research@nremt.org. If you have concerns or questions about your rights as a participant, you can contact the Chair of AIR's Institutional Review Board (which is responsible for the protection of study participants) at IRBChair@air.org, toll-free at 1-800-634-0797 or c/o AIR, 1000 Thomas Jefferson Street, NW, Washington, DC 20007.

Please click 'Next' to continue.

1. To go back to a previous question, use the back arrow at the bottom of the page. Do NOT use your browser's back arrow.
2. If you want to take a break, you can save your answers using the save button at the top of your screen. You can return to the survey by clicking on the URL in the email invitation you received.

How many years have you worked as an EMS professional?

- I have never worked as an EMS professional
- Less than one year
- 1-2 years
- 3-4 years
- 5-7 years
- 8-10 years
- 11-15 years
- 16-20 years
- 21 or more years

For how many different organizations do you currently perform EMS work?

- 0
- 1
- 2 or more

Which of the following best describes your <u>primary role</u> at your main EMS job?

- *Patient Care Provider* - A person whose primary role is the provision of EMS services to patients.
- *Educator* - A person whose primary role is instructing individuals enrolled in an approved or accredited EMS training course or providing continuing education required for maintenance of licensure.
- *Preceptor* - A person whose primary role is training individuals enrolled in an approved or accredited EMS training course in a clinical setting.

○ *Dispatcher/Call Taker* - A person whose primary role is EMS communications.

○ *Administrator/Manager* - A person whose primary role is the management and direction of an organization providing EMS services.

○ *First-line Supervisor* - A person whose primary role is the direct supervision of individuals providing EMS services.

○ *Other* - A person whose primary EMS role at their main job is not listed above (please specify).: ______

Which of the following best describes your main EMS agency/organization?

○ *Hospital* - refers to EMS agencies that are under the direct control of a hospital, regardless of the type of organization that runs the hospital.

○ *Fire Department* - an organization from which fire and EMS services are provided, regardless of the type of organization that runs the Fire Department. Volunteer fire departments should be included here.

○ *Tribal* - are operated by a federally recognized Indian or Alaska Native Tribe.

○ *Military* - are operated by one of the U.S. Armed Forces and staffed by active duty personnel.

○ *Government, Non-Fire Department* - are operated directly by a federal, state, county, or local government entity other than the U.S. Armed Forces.

○ *Private* - are operated under the direct control of a for-profit or not-for-profit organization other than a hospital. Volunteer rescue squads that are operated independently of a fire department should be included here.

○ *Air Medical* - an organization which provides air ambulance services, regardless of the type of organization which runs the air ambulance service.

○ Other - Please specify: ______

Which of the following best describes the primary type of service provided by your main EMS agency/organization? ***If more than one type of service is provided, pick the service with the greatest number of calls in the past 12 months.***

○ *Primarily 911 response with or without transport capability* - Immediate response to an incident location, regardless of method of notification (for example, 911, direct dial, walk-in, flagging down).

○ *Primarily medical transport (convalescent)* - Transport of a patient from one health facility to another.

○ *Equal mix of 911 and medical transport (convalescent)*

○ *Clinical services* - Provision of clinical services in an non-ambulance clinical setting such as emergency department, medical office, or dialysis clinic.

○ *Mobile Integrated Healthcare & Community Paramedicine* - Provision of clinical services in an out-of-hospital community setting.

○ *Other* - Please specify: ______

In the past 12 months, have you provided any patient care in the prehospital setting?

○ Yes

○ No

The following questions ask about your experience with identifying, opening and administering <u>medications</u>.

Please consider all forms of medications you have administered in the prehospital setting in the past 12 months.

In the past 12 months, have you had difficulty <u>identifying</u> a medication while providing care in the prehospital setting?

○ Yes

○ No

In the past 12 months, which of the following has made it difficult for you to identify a medication?

- [ ] Lack of transparency of package made it difficult to tell what product was
- [ ] Crowded label made it difficult to read
- [ ] Small text on label made it difficult to read
- [ ] Different medications have similar packaging
- [ ] Confusing names
- [ ] Dark conditions made it difficult to read labels
- [ ] Other - Please describe: ____________

In the past 12 months, which of the following have you used to cope when medications were difficult to identify?

- [ ] Flashlight
- [ ] Touch/feel
- [ ] Changed the location of product within container, bag or ambulance
- [ ] Other - Please describe: ____________

In the past 12 months, has an issue with identifying a medication negatively impacted your patient care?

- ○ Yes
- ○ No

In the past 12 months, have you had difficulty <u>opening</u> a medication?

- ○ Yes
- ○ No

In the past 12 months, which of the following has made it difficult for you to open a medication?

- [ ] Too small of an area to grip
- [ ] Materials meant to separate stuck together
- [ ] Product required too much force to open
- [ ] Product required two hands to open
- [ ] Unfamiliar with product packaging
- [ ] Packaging directions for opening were not clear
- [ ] Other - Please describe: ____________

In the past 12 months, which of the following have you used to cope when medications were difficult to open?

- [ ] Knives
- [ ] Scissors
- [ ] Teeth
- [ ] Pen
- [ ] Partner assistance
- [ ] Other - Please describe: ____________

In the past 12 months, has an issue with opening a medication negatively impacted your patient care?

- ( ) Yes
- ( ) No

In the past 12 months, have you had difficulty <u>administering</u> a medication?

- ○ Yes
- ○ No

In the past 12 months, which of the following has made it difficult for you to administer a medication?

- ☐ Medication characteristics made it difficult to remove
- ☐ Product stuck to package
- ☐ Complicated packaging features
- ☐ Vehicle (e.g., ambulance) movement and vibration
- ☐ Other - Please describe: ______

In the past 12 months, has an issue with administering a medication negatively impacted your patient care?

- ○ Yes
- ○ No

The following questions ask about your experience with identifying, opening and using ***medical supplies*** (e.g., syringes, endotracheal tubes, IV administration sets, etc.).

Please consider all forms of medical supplies you have used in the prehospital setting in the past 12 months.

In the past 12 months, have you had difficulty <u>identifying</u> a medical supply (e.g., syringe, endotracheal tube, IV administration set) while providing care in the prehospital setting?

- ○ Yes

No

In the past 12 months, which of the following has made it difficult for you to identify medical supplies?

- Lack of transparency of package made it difficult to tell what product was
- Crowded label made it difficult to read
- Different supplies had similar packaging
- Confusing names
- Dark conditions made it difficult to read labels
- Other - Please describe:

In the past 12 months, which of the following have you used to cope when medical supplies were difficult to identify?

- Flashlight
- Touch/feel
- Changed the location of product within container, bag or ambulance
- Other - Please describe:

In the past 12 months, has an issue with identifying medical supplies negatively impacted your patient care?

Yes

No

In the past 12 months, have you had difficulty <u>opening</u> medical supplies (e.g., a syringe, endotracheal tube, or IV administration set)?

- Yes
- No

In the past 12 months, which of the following has made it difficult for you to open medical supplies?

- Too small of an area to grip
- Materials meant to separate stuck together
- Product opened with too much force
- Product required two hands to open
- Unfamiliar with product packaging
- Packaging directions for opening were not clear
- Other - Please describe:

In the past 12 months, which of the following have you used to cope when medical supplies were difficult to open?

- Knives
- Scissors
- Teeth
- Pen
- Partner assistance
- Other - Please describe:

In the past 12 months, has an issue with opening medical supplies negatively impacted your patient care?

- Yes
- No

In the past 12 months, have you had difficulty <u>using</u> a medical supply?

- Yes
- No

In the past 12 months, which of the following has made it difficult for you to use a medical supply?

- Product characteristics made it difficult to remove
- Product stuck to package
- Multiple layers of packaging
- Multiple, loose items
- Other - Please describe: ______

In the past 12 months, has an issue with using a medical supply negatively impacted your patient care?

- Yes
- No

How long have you been employed or volunteered at your main EMS job?

- Less than one year
- 1-2 years
- 3-4 years

- 5-7 years
- 8-10 years
- 11-15 years
- 16-20 years
- 21 or more years

Which of the following best describes your employment status at your main EMS job?

- Full time
- Part time
- Per diem, PRN or as needed
- Volunteer or on-call

On average, how many calls do you respond to in a typical week at your main EMS job?

- 0
- 1
- 2 to 4
- 5 to 9
- 10 to 19
- 20 to 29
- 30 to 39
- 40 to 49
- 50 or more

Which of the following best describes the community in which you do most of your EMS work?

- Rural area (less than 2,500 people)
- Small town (2,500 - 24,999 people)
- Medium town (25,000 -74,999 people)
- Large town (75,000 - 149,999 people)
- Mid-sized city (less than 500,000 people)
- Suburb/fringe of a mid-sized city
- Large city (500,000 or more people)
- Suburb/fringe of a large city

The following section asks questions about you. This information will be used for classification purposes only and the data you share will not be used to personally identify you. You may choose not to answer any question by clicking 'Next.'

In what year were you born?

What is the highest level of education you have completed?

- Didn't complete high school
- High school graduate/GED
- Some college
- Associate's Degree
- Bachelor's Degree
- Master's Degree

○ Doctoral Degree

What is your sex?

○ Male

○ Female

Are you Hispanic or Latino?

○ Yes

○ No

○ Refuse

Which of the following best describes you? You may choose more than one.

☐ American Indian or Alaskan Native

☐ Asian

☐ Black or African American

☐ Native Hawaiian or other Pacific Islander

☐ White

☐ Refuse

Thank You!

Thank you for completing this survey. Your response is very important to us and will help shape our profession!

If you have any questions, or want to obtain more information about this very important project, please contact the NREMT Research Department at 614-888-4484 or via email at research@nremt.org.
